# Progressive pulmonary fibrosis in myositis-specific antibody-positive interstitial pneumonia: a retrospective cohort study

**DOI:** 10.3389/fmed.2023.1325082

**Published:** 2024-01-11

**Authors:** Huijuan Wang, Yuanying Wang, Di Sun, Shiwen Yu, Xuqin Du, Qiao Ye

**Affiliations:** ^1^Clinical Center for Interstitial Lung Diseases, Beijing Institute of Respiratory Medicine, Beijing Chao-Yang Hospital, Capital Medical University, Beijing, China; ^2^Department of Occupational Medicine and Toxicology, Beijing Chao-Yang Hospital, Capital Medical University, Beijing, China

**Keywords:** myositis-specific antibody, progressive pulmonary fibrosis, predictors, antifibrotic drugs, survival

## Abstract

**Objectives:**

Idiopathic inflammatory myopathy (IIM) frequently coexists with interstitial pneumonia (IP) and is commonly the initial or sole manifestation accompanied by positive myositis-specific autoantibodies (MSAs), even in the absence of meeting diagnostic criteria. This study aims to evaluate the proportion of progressive pulmonary fibrosis (PPF) and identify potential predictors influencing the progression of pulmonary fibrosis in patients with MSA-IP.

**Methods:**

This descriptive study employed a retrospective cohort design, enrolling patients diagnosed with interstitial pneumonia and positive MSAs at Beijing Chao-Yang Hospital in a sequential manner. Clinical data were systematically collected from the patients’ medical records during regular follow-up visits conducted every 3 to 6 months. Cox regression analysis was utilized to identify independent predictors of PPF in patients with positive MSAs and interstitial pneumonia.

**Results:**

A total of 307 patients were included in the study, with 30.6% of them developing PPF during a median follow-up period of 22 months. Kaplan–Meier survival curves demonstrated a significantly lower survival in the PPF patients compared to the non-PPF patients (median 11.6 months vs. 31 months, *p* = 0.000). An acute/subacute onset of interstitial pneumonia (HR 3.231, 95%CI 1.936–5.392, *p* = 0.000), lower diffusing capacity of the lungs for carbon monoxide (DLCO) % predicted (HR 6.435, 95%CI 4.072–10.017, *p* = 0.001), and the presence of diffuse alveolar damage (DAD) on high-resolution computed tomography (HRCT) (HR 8.679, 95%CI 1.974–38.157, *p* = 0.004) emerged as independent predictors of PPF. Notably, the implementation of triple therapy comprising glucocorticoids, immunosuppressants, and antifibrotic drugs was associated with a reduced risk of developing PPF (HR 0.322, 95%CI 0.115–0.899, *p* = 0.031).

**Conclusion:**

Approximately 30.6% of patients with MSA-IP may develop PPF within the follow-up period. Patients presenting with an acute/subacute onset of interstitial pneumonia, lower predicted DLCO SB% and evidence of DAD on HRCT are more susceptible to developing PPF. Conversely, the administration of triple therapy appears to serve as a protective factor against the development of PPF in patients with MSA-IP.

## Introduction

Idiopathic inflammatory myopathies (IIMs) comprise a diverse group of systemic autoimmune diseases, including polymyositis (PM), dermatomyositis (DM), antisynthetase syndrome, immune-mediated necrotizing myositis, connective tissue-associated myositis (overlapping myositis), and sporadic inclusion body myositis (1). These conditions involve varying degrees of skin, muscle, and lung involvement. Interstitial lung disease (ILD) is a frequent pulmonary manifestation and a significant cause of morbidity and mortality in patients with IIMs (2). Accurate diagnosis of ILD is crucial in patients with IIM or other connective tissue diseases (CTDs) due to its diverse presentations.

Myositis-specific antibodies (MSAs) have gained increasing attention for their role in distinguishing various types of IIMs and their association with the risk of developing ILD (3, 4). Different MSAs, including anti-aminoacyl tRNA synthetase (anti-ARS) antibodies, anti-melanoma differentiation-associated gene 5 (MDA5) antibodies, and other rare antibodies, have been identified. MSAs are relevant to the pathogenesis and prognosis of patients with IIM, with specific antibodies linked to clinical subtypes and increased risk of ILD (3, 4). In cases where patients with interstitial pneumonia exhibit features suggestive of underlying autoimmune conditions but do not meet diagnostic criteria for any specific CTD, a diagnosis of interstitial pneumonia with autoimmune features may be considered (5).

Consensus has been emerging regarding the treatment of interstitial lung disease associated with idiopathic inflammatory myopathies (IIM-ILD) (6). The current approach involves combining glucocorticoids with immunosuppressive agents as first-line treatment, including mycophenolate, cyclosporine, tacrolimus, cyclophosphamide, azathioprine, hydroxychloroquine, and others (6). High-resolution computed tomography (HRCT) plays a crucial role in the assessment of fibrotic manifestations in IIM-ILD patients (7). However, despite early initiation of treatment, pulmonary fibrosis often progresses, leading to a poorer prognosis. Antifibrotic drugs such as pirfenidone or nintedanib are frequently added to slow down the rate of progression in this subset of patients. Several factors have been identified as predictors of a poor prognosis in patients with IIM-ILD, including acute onset of ILD, lower forced vital capacity (FVC) % predicted at ILD onset, positive anti-Ro-52 antibody, positive anti-MDA5 antibody, and the presence of usual interstitial pneumonia (UIP) (8–11). The clinical characteristics of progressive fibrosis in patients with myositis-specific antibody-positive interstitial pneumonia (MSA-IP) have been described by clustering in our previous studies and demonstrated that anti-MDA5 positive and acute or subacute of ILD patients were prone to interstitial pneumonia progression (12). However, there is limited data on progressive pulmonary fibrosis (PPF) in patients with MSA-IP, and the factors influencing the progression of pulmonary fibrosis in these patients have not been well elucidated. Therefore, this study aimed to investigate the incidence, clinical characteristics, and risk factors associated with PPF in MSA-IP patients through survival analysis.

## Methods

### Study design and patient selection

This was a retrospective cohort study conducted at Beijing Chaoyang Hospital, a regional tertiary referral center specializing in ILD. Patients aged 18 years or older, diagnosed with interstitial pneumonia through chest HRCT and exhibiting positive MSAs, were screened in a retrospective manner from January 2017 to June 2022. All patients with interstitial pneumonia were diagnosed according to the 2013 American Thoracic Society and European Respiratory Society consensus classification criteria for idiopathic interstitial pneumonia (13). Multidisciplinary diagnoses were conducted between pulmonologists, radiologists, rheumatologists, and pathologists experienced in the diagnosis of ILD based on clinical characteristics, HRCT, and lung biopsy if appropriate. Patients with any of the following reasons were excluded: (1) lack of MSA test or with negative MSAs; (2) interstitial pneumonia of known etiology, such as sarcoidosis, pneumoconiosis, drugs, radiation, gastroesophageal reflux disease-associated interstitial pneumonia; and (3) combined with severe underlying diseases such as chronic obstructive pulmonary disease, asthma, pulmonary infectious diseases, heart failure. The Ethics Committee of Beijing Chaoyang Hospital approved this study, and all procedures were performed in accordance with the principles of the Declaration of Helsinki.

### Data collection

The medical records of the patients were reviewed to extract clinical information from their initial visit. This information included demographic data (age, gender, smoking history, height, and weight), symptoms and signs (respiratory and extrapulmonary multisystem involvement), laboratory data, parameters of pulmonary function tests, chest HRCT images, and therapy regimens.

The laboratory data encompassed blood cell count and derivative blood cell count inflammation indexes, C-reactive protein, erythrocyte sedimentation rate, immunoglobulin levels, various autoantibodies, and MSA subtypes. MSAs include anti-ARS antibodies, anti-MDA5 antibody, anti-signal recognition particle (SRP) antibody, anti-Mi-2 antibody, anti-small ubiquitin-like modifier activating enzyme (SAE) antibody, anti-nuclear matrix protein (NXP) 2 antibody and anti-transcriptional intermediary factor (TIF)-γ antibody, of which anti-ARS antibodies include anti-histidyl-tRNA synthetase (Jo-1) antibody, anti-threonyl-tRNA synthetase (PL-7) antibody, anti-alanyl-tRNA synthetase (PL-12) antibody, anti-isoleucyl-tRNA synthetase (OJ) antibody, anti-glycyl-tRNA synthetase (EJ) antibody. MSAs were detected qualitatively using an immunoblotting method by using a reagent kit provided by Shenzhen Avalon Biotechnology Co. Ltd. and were operated strictly according to the instructions (Supplementary material).

The oxygenation index, calculated as the ratio of arterial partial pressure of oxygen to inhaled oxygen concentration, was also recorded. Additional methodological details can be found in the Supplementary material.

### HRCT scans

All patients underwent chest HRCT scans with a 1-s scan time, 0.625-mm sections, and 10-mm intervals from the lung apex to the base including both lungs in the field of view, and the imaging results were evaluated by radiologists. The radiological patterns observed on HRCT were consistent with international idiopathic interstitial pneumonia classifications, including non-specific interstitial pneumonia (NSIP), organizing pneumonia (OP), NSIP overlapping with OP, UIP, and diffuse alveolar damage (DAD). Please refer to the Supplementary material for some definitions.

### Follow-up and study ending

The follow-up period ended on June 2022. Follow-up information was collected via medical records every 3–6 months. The primary outcome was PPF. Patients with ILD other than idiopathic pulmonary fibrosis (IPF) with radiological evidence of pulmonary fibrosis who fulfilled at least two of the following criteria in the past year, and in the absence of other explanations were considered PPF (14): (1) worsening respiratory symptoms; (2) physiological evidence (either of the following): a. absolute decline in FVC >5% predicted within 1 year of follow-up b. absolute decline in DLCO (corrected for Hb) >10% predicted within 1 year of follow-up; and (3) radiological evidence: a. increased extent or severity of traction bronchiectasis and bronchiectasis; b. new ground-glass opacity with traction bronchiectasis; c. new fine reticulation; d. increased extent or increased coarseness of reticular abnormality; e. new or increased honeycombing; f. increased lobar volume loss. The secondary outcome was all-cause mortality during the follow-up period or the end of follow-up. Survival time was calculated from the time of the first diagnosis to the outcome or the end of follow-up.

### Statistical analysis

Data analysis was performed using the statistical software SPSS 26.0 and Origin 2022 for graphing. Normally distributed measures were presented as mean ± standard deviation (SD), and data analysis was conducted using *t*-tests or ANOVA. Non-normally distributed measures were expressed as a median or interquartile range and non-parametric tests were employed for data analysis. Count data were reported as the number of cases (percentage), and Chi-square tests or Fisher’s exact probability method were used for data analysis. Cox proportional hazards model was utilized to analyze factors influencing patients’ prognosis. A significance level of *p* < 0.05 was considered statistically significant.

## Results

### Study population

**Figure 1:**
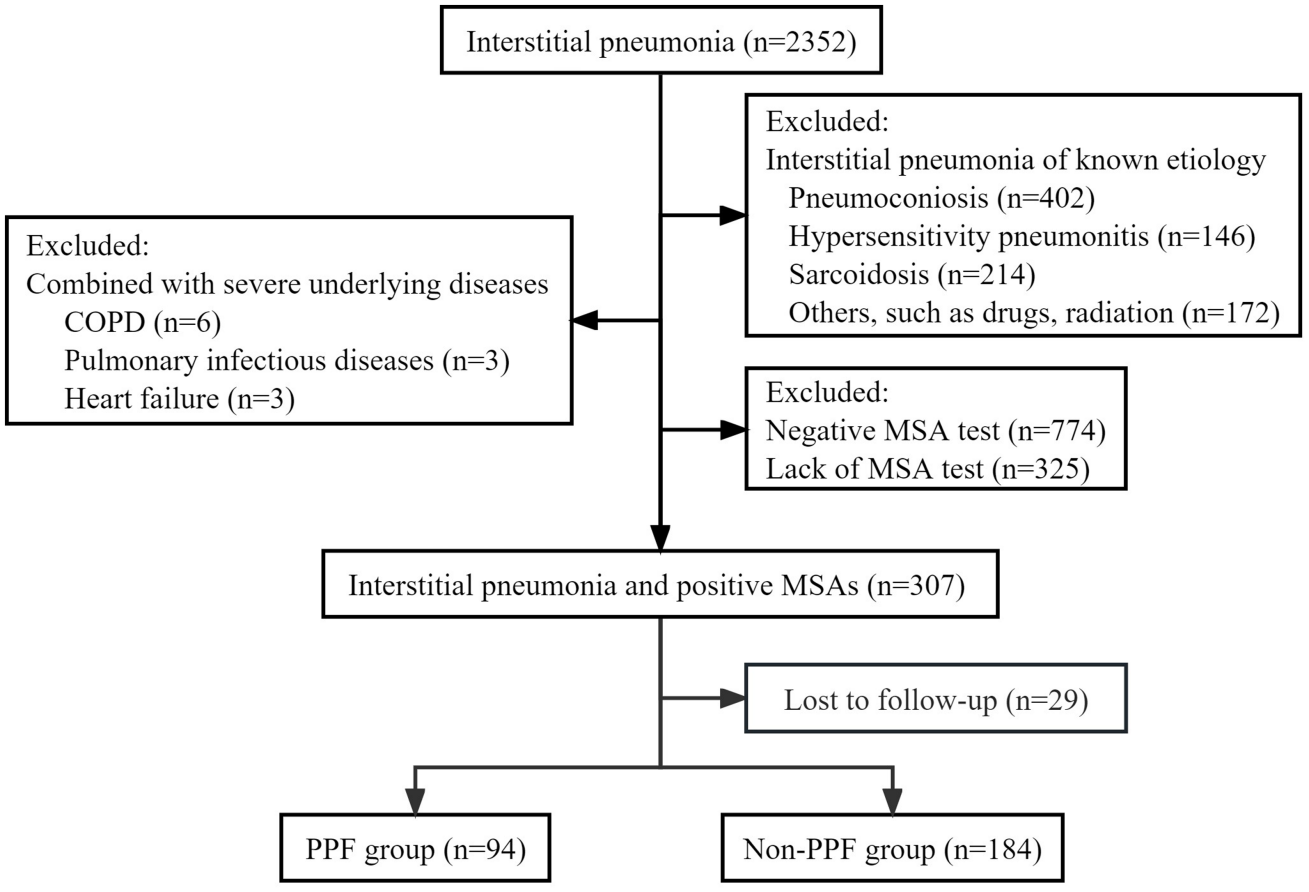
Flow chat of screening the study population. COPD, chronic obstructive pulmonary disease; MSA, myositis-specific antibody; PPF, progressive pulmonary fibrosis.

During the study period, a consecutive screening of patients diagnosed with interstitial pneumonia based on chest HRCT was conducted, including both inpatients and outpatients attending the clinic. After excluding factors such as negative MSA or lack of MSA test results, a total of 307 patients were included in the final analysis, comprising 94 (30.62%) PPF patients and 213 (69.38%) non-PPF patients. The detailed flow diagram depicting the patient selection process is illustrated in Figure 1.

### Demographics and clinical characteristics

In the entire cohort, the mean (±SD) age was 58.6 (±11.3) years, with females constituting 64.5% of the patient population. Among the patients, 74.3% were non-smokers. Approximately 56.4% of patients with PPF experienced an acute/subacute onset of interstitial pneumonia, while the majority of non-PPF patients exhibited a chronic onset. Dyspnea was the prevailing respiratory symptom among PPF patients (*p* < 0.01), and baseline measurements including oxygenation index (*p* < 0.05), FVC% pred. (*p* < 0.05), and diffusing capacity of the lung for carbon monoxide (DLCO) SB% pred. (*p* < 0.05) were significantly lower in comparison to non-PPF patients. In terms of extra-pulmonary organ system involvement, except for dry eyes, dry mouth, and rampant caries (*p* < 0.05), no statistically significant differences were observed in other symptoms, including proximal muscle weakness, skin rash, Gottron’s sign, mechanic’s hand, photosensitivity, and arthralgia (Table 1).

**Table 1:** Demographics and clinical characteristics of the enrolled patients.

Variables	All	PPF	Non-PPF	T/U/χ^2^	*p*-value
*N*	307	94	213		
Age, years	58.6 ± 11.3	60.3 ± 11.8	57.8 ± 11.0	1.785	0.194
Female, *n* (%)	198 (64.5)	54 (57.4)	144 (67.6)	2.939	0.086
Smoking status, *n* (%)				3.414	0.181
Current	35 (11.4)	15 (16.0)	20 (9.4)		
Former	44 (14.3)	15 (16.0)	29 (13.6)		
Never	228 (74.3)	64 (68.1)	164 (77.0)		
BMI, Kg/m^2^, *n* (%)	211	70	141	2.239	0.326
<18.5	6 (2.8)	3 (4.3)	3 (2.1)		
18.5–24.9	110 (52.1)	40 (57.1)	70 (49.6)		
≥25.0	95 (45.0)	27 (38.6)	68 (48.2)		
ILD onset, *n* (%)				12.025	0.001
Acute/subacute	128 (41.7)	53 (56.4)	75 (35.2)		
Chronic	179 (58.3)	41 (43.6)	138 (64.8)		
Cough, *n* (%)	254 (82.7)	80 (85.1)	174 (81.7)	0.533	0.465
Dyspnea, *n* (%)	251 (81.8)	85 (90.4)	166 (77.9)	6.823	0.009
PAH, *n* (%)	18 (6.1)	7 (7.7)	11 (5.4)	0.565	0.452
PaO_2_ / FiO_2_, mmHg (IQR)	280.7 (241.5–312.0)	251.4 (220.3–293.4)	291.4 (247.4–319.8)	448.000	0.041
CPI (SD)	37.2 ± 16.7	43.2 ± 17.0	34.4 ± 15.9	−3.816	0.835
Pulmonary functions (SD)					
FVC, L	2.39 ± 0.77	2.27 ± 0.81	2.44 ± 0.75	1.559	0.375
FVC, % pred.	80.44 ± 20.78	64.25 ± 16.73	82.00 ± 20.28	6.439	0.013
DLCO SB, % pred.	59.54 ± 18.66	41.42 ± 13.29	62.74 ± 18.41	8.740	0.022
Fever, *n* (%)	81 (26.4)	29 (30.9)	52 (24.4)	1.392	0.238
Proximal musculature weakness, *n* (%)	29 (9.4)	7 (7.4)	22 (10.3)	0.633	0.426
Dysphagia, *n* (%)	7 (2.3)	0 (0.0)	7 (3.3)	3.161	0.075
Skin rash, *n* (%)	59 (19.2)	17 (18.1)	42 (19.7)	0.112	0.738
Gottron’s sign, *n* (%)	23 (7.5)	7 (7.4)	16 (7.5)	0.000	0.984
Mechanic’s hands, *n* (%)	38 (12.4)	7 (7.5)	31 (14.6)	2.939	0.086
Photosensitivity, *n* (%)	22 (7.2)	3 (3.2)	19 (8.9)	3.217	0.093
Fingertip hardening, *n* (%)	9 (2.9)	3 (3.2)	6 (2.8)	0.032	1.000
Joint pain, *n* (%)	81 (26.4)	22 (23.4)	59 (27.7)	0.619	0.431
Joint swelling, *n* (%)	18 (5.9)	5 (5.3)	13 (6.1)	0.073	0.788
Morning stiffness, *n* (%)	23 (7.5)	8 (8.5)	15 (7.0)	0.203	0.652
Raynaud’s phenomenon, *n* (%)	12 (3.9)	5 (5.3)	7 (3.3)	0.717	0.397
Vasculitis, *n* (%)	3 (1.0)	2 (2.1)	1 (0.5)	1.853	0.223
Dry eye, *n* (%)	56 (18.2)	27 (28.7)	29 (13.6)	9.982	0.002
Dry mouth, *n* (%)	74 (24.1)	34 (36.2)	40 (18.8)	10.782	0.001
Rampant caries, *n* (%)	15 (4.9)	10 (10.6)	5 (2.3)	9.647	0.002
Hair loss, *n* (%)	14 (4.6)	6 (6.4)	8 (3.8)	1.034	0.309
Mouth ulcers, *n* (%)	10 (3.3)	5 (5.3)	5 (2.3)	1.828	0.176
Gastroesophageal reflux, *n* (%)	16 (5.2)	4 (4.3)	12 (5.6)	0.251	0.783

Data was presented as mean ± SD or *n* (%). MSA, myositis-specific antibodies; IP, interstitial pneumonia; PPF, progressive pulmonary fibrosis; BMI, body mass index; ILD, interstitial lung disease; PAH, pulmonary hypertension; CPI, composite physiologic index; FVC, forced vital capacity; DLCO, diffusing capacity of the lung for carbon monoxide; SD, standard deviation.

### Laboratory data and MSA subtypes

All patients underwent comprehensive laboratory tests, including complete blood count and autoantibody assessments. 41.7% of enrolled patients were anti-Ro-52 positive. No statistically significant differences were found between PPF patients and non-PPF patients on anti-Ro-52. Similarly, there were no statistically significant differences observed in baseline blood cell counts, derivative blood cell count inflammatory indexes, and immunoglobulin levels between the two groups (Table 2).

**Table 2:** Laboratory findings of the enrolled patients.

Variables	All	PPF	Non-PPF	T/U/χ^2^	*p*-value
*N*	307	94	213		
Blood count values (IQR)					
WBC (×10^9^ /L)	7.41 (5.48–9.43)	7.62 (6.20–9.93)	7.12 (5.31–9.35)	8050.5	0.089
Neutrophils (×10^9^ /L)	4.66 (3.26–7.14)	5.20 (3.63–7.68)	4.50 (3.23–7.68)	8159.5	0.124
Lymphocytes (×10^9^ /L)	1.52 (1.02–2.14)	1.42 (1.00–2.20)	1.55 (1.04–2.20)	8887.5	0.651
Monocyte (×10^9^ /L)	0.44 (0.34–0.59)	0.47 (0.35–0.57)	0.44 (0.33–0.59)	8768.5	0.529
RDW	13.20 (12.5–14.15)	13.20 (12.60–14.20)	13.20 (12.40–14.13)	8862.5	0.624
MLR	0.30 (0.20–0.43)	0.32 (0.21–0.49)	0.29 (0.19–0.41)	8170.5	0.128
NLR	2.95 (1.99–5.78)	3.61 (2.06–6.63)	2.77 (1.92–5.00)	8103.0	0.105
PLR	160.57 (106.85–227.68)	150.78 (101.01–239.32)	165.43 (107.02–220.25)	8919.5	0.686
SIRI	1.33 (0.78–2.72)	1.46 (0.89–3.49)	1.28 (0.75–2.49)	7958.5	0.066
AISI	329.58 (156.83–670.52)	364.14 (170.85–807.04)	306.99 (150.84–636.63)	8463.5	0.278
CRP, *n* (%)	120 (39.1)	40 (42.6)	80 (37.6)	1.623	0.654
ESR, mm/1 h (SD)	22.12 ± 18.05	23.31 ± 14.91	21.67 ± 19.36	0.648	0.169
Fibrinogen, mg/dL (SD)	342.45 ± 113.71	361.8 ± 118.84	332.53 ± 110.06	−1.870	0.295
IgG, mg/dL	1300.00 (1067.50–1610.00)	1285.00 (1015.00–1667.50)	1280.00 (1060.00–1520.00)	7001.50	0.926
IgA, mg/dL	277.00 (194.00–358.50)	286.00 (198.50–377.75)	271.00 (193.00–337.00)	5901.50	0.237
IgM, mg/dL	110.00 (74.40–162.00)	115.00 (72.475–168.75)	106.00 (75.70–162.00)	6260.00	0.732
ANA, *n* (%)	133 (48.9)	35 (41.2)	98 (52.4)	2.949	0.086
Anti-dsDNA antibody, *n* (%)	1 (0.4)	0 (0.0)	1 (0.6)	0.486	1.000
MPO-ANCA, *n* (%)	4 (1.6)	3 (3.9)	1 (0.6)	3.734	0.088
PR3-ANCA, *n* (%)	4 (1.6)	3 (3.9)	1 (0.6)	3.831	0.085
RF, *n* (%)	20 (8.9)	9 (13.8)	11 (6.9)	2.723	0.099
Anti-CCP antibody, *n* (%)	16 (7.2)	8 (11.8)	8 (5.2)	2.995	0.084
Anti-SSA antibody, *n* (%)	99 (39.3)	26 (33.8)	73 (41.7)	1.416	0.234
Anti-SSB antibody, *n* (%)	10 (4.1)	6 (8.2)	4 (2.3)	4.500	0.070
Anti-Ro-52 antibody, *n* (%)	128 (41.7)	40 (42.6)	88 (41.3)	0.041	0.839
Anti-PM-Scl antibody, *n* (%)	15 (4.9)	5 (5.3)	10 (4.7)	0.055	0.815
Anti-U1-snRNP antibody, *n* (%)	9 (2.9)	1 (1.1)	8 (3.8)	1.661	0.284

Data were presented as *n* (%) or median (IQR). WBC, white blood cell; RDW, red cell distribution width; MLR, monocyte to lymphocyte ratio; NLR, neutrophil to lymphocyte ratio; PLR, platelet to lymphocyte ratio; SIRI, systemic inflammatory response index; AISI, aggregate index of systemic inflammation; CRP, C-reactive protein; ESR, erythrocyte sedimentation rate; Ig, immune globulin; ANA, antinuclear antibody; dsDNA, double-stranded DNA; MPO-ANCA, myeloperoxidase-antineutrophil cytoplasmic antibody; PR3-ANCA, anti-proteinase 3-antineutrophil cytoplasmic antibody; RF, rheumatoid factor; CCP, cyclic citrullinated peptide; U1-snRNP, U1 small nuclear ribonucleoprotein.

Regarding MSA subtypes, among the 307 patients, 64.8% tested positive for anti-ARS antibodies, while 35.2% were positive for non-ARS MSAs. The positive rates of anti-Jo-1, anti-PL-7, anti-EJ, and anti-MDA5 antibodies were higher compared to other antibodies, with rates of 21.8, 15.0, 16.6, and 18.6%, respectively. Among the anti-ARS antibodies, anti-PL-7 and anti-OJ antibodies showed slightly higher positivity in PPF patients, while anti-Jo-1 positivity was lower compared to non-PPF patients (*p* < 0.05). In this study, we found that 6.5% (20/307) of patients were anti-Mi-2β positive, while only 1.6% (5/307) were anti-Mi-2α antibody positive (with 4 in the PPF patients and 1 in the non-PPF patients). Although there was a significant difference in anti-Mi-2α between PPF and non-PPF patients (*p* < 0.05), the small number of individuals in this group led us to consider the results not clinically significant. However, the difference in anti-MDA5 between the two groups was not statistically significant (Supplementary Table S1).

### Chest HRCT imaging characteristics

All enrolled patients underwent chest HRCT, and the imaging findings were independently evaluated by two radiologists with a kappa coefficient of 0.78. The identified imaging characteristics included ground glass opacity, solid shadow, honeycombing opacity, reticular opacity, and traction bronchiectasis. HRCT patterns such as NSIP, OP, NSIP+OP, and UIP were observed in both PPF and non-PPF patients. The distribution of NSIP, UIP, and unclassifiable interstitial pneumonia patterns was similar between the two groups, accounting for 40.4% vs. 41.3, 19.1% vs. 16.4, and 5.3% vs. 6.6%, respectively. All 11 DAD patterns were observed in the PPF patients. Over 60% of patients with DAD were caused by an inflammatory, and during the follow-up period, fibrotic manifestations such as reticulation and traction bronchiectasis gradually appear (Supplementary Table S2).

### Treatments

Among the 307 enrolled patients, 54 received glucocorticoids alone, 172 were treated with glucocorticoids in combination with immunosuppressive agents, and 31 received triple therapy consisting of glucocorticoids, immunosuppressive agents, and anti-fibrotic agents. The patients receiving immunosuppressive agents were further categorized based on the specific agents used, including mycophenolate, cyclosporine, tacrolimus, cyclophosphamide, azathioprine, methotrexate, and hydroxychloroquine. Cyclophosphamide was the most frequently prescribed agent, followed by hydroxychloroquine and tacrolimus. Additionally, 14.5% of patients received a combination of two or more immunosuppressive agents. In this study, when treating patients who developed pulmonary fibrosis, the doctors made decisions about administering antifibrotic drug based on the patients’ preferences and their family’s financial support at baseline. During follow-up, the indication for anti-fibrotic treatment (pirfenidone and nintedanib) in this study was PPF. Pirfenidone was significantly more commonly used than nintedanib (83.9% vs. 16.1%, *p* = 0.065). Out of the 31 patients who received triple therapy (including antifibrotic drugs) at baseline, 5 patients experienced disease progression, while 26 patients did not during follow-up. However, there were no statistically significant differences in the frequency of immunosuppressive and anti-fibrotic agent use between the two groups of patients (Table 3). Tofacitinib was administered to two patients, and intravenous immunoglobulin was given to two patients; none of these four patients showed progression of fibrosis. Only 4 patients were diagnosed with Pneumocystis jirovecii pneumonia, and the majority of patients had no evidence of Pneumocystis jirovecii infection, but 4.5% of enrolled patients received sulfonamide drugs for the prevention of Pneumocystis jirovecii pneumonia.

**Table 3:** Therapeutic regimens of the enrolled patients.

Variables	All	PPF	Non-PPF	T/U/χ^2^	*p*-value
N	307	94	213		
Corticosteroids, *n* (%)	54 (17.6)	17 (18.1)	37 (17.4)	0.023	0.880
Corticosteroids + immunosuppressants, *n* (%)	172 (56.0)	58 (61.7)	114 (53.5)	1.772	0.183
+Mycophenolate	22 (12.8)	8 (13.8)	14 (12.3)	0.079	0.779
+Cyclosporine	2 (1.2)	0 (0)	2 (1.8)	1.030	0.550
+Tacrolimus	30 (17.4)	5 (8.6)	25 (21.9)	4.729	0.030
+Cyclophosphamide	97 (56.4)	36 (62.1)	61 (53.5)	1.146	0.284
+Azathioprine	6 (3.5)	2 (3.4)	4 (3.5)	0.000	1.000
+Methotrexate	5 (2.9)	1 (1.7)	4 (3.5)	0.434	0.664
+Hydroxychloroquine	38 (22.1)	17 (29.3)	21 (18.4)	2.648	0.104
≥ 2	25 (14.5)	9 (16.7)	16 (13.6)	0.288	0.592
Triple therapies^a^, *n* (%)	31 (10.1)	5 (5.3)	26 (12.2)	3.408	0.065
+Pirfenidone	26 (83.9)	5 (100)	21 (80.8)	1.146	0.284
+Nintedanib	5 (16.1)	0 (0)	5 (19.2)	1.146	0.560
Other therapies	50 (16.3)	14 (14.9)	36 (16.9)	0.248	0.969

Data were presented as *n* (%).

a Triple therapies include corticosteroids, immunosuppressants, and anti-fibrotic agents.

### Survival and risk factors for PPF

The time interval from diagnosis of myositis to PPF is 0.9 to 12 months (median time of 7.5 months). The patients in the entire cohort were followed for a median duration of 22 months, ranging from 0.9 to 66 months. 75.6% (232/307) of enrolled patients had a baseline chest HRCT showing fibrotic ILD, and among PPF patients, 80.9% (76/94) of patients had baseline chest HRCT showing fibrotic ILD. Among both the PPF and non-PPF patients during the follow-up period, there were instances of mortality, with 8 deaths occurring in the PPF patients and 6 deaths in the non-PPF patients. The cause of death among the PPF patients was respiratory failure due to rapidly progressive interstitial lung disease (RPILD), while two of the non-PPF patients died from septic shock and four died from respiratory failure.

As shown in Table 4, in the univariate Cox regression analysis, risk factors associated with the presence of PPF included elderly individuals, acute/subacute onset of interstitial pneumonia, ANCA-positive, positive anti-CCP antibody, SIRI, lower baseline FVC %pred, lower baseline DLCO SB %pred, and HRCT showing DAD. The use of triple therapy (including anti-fibrotic drugs) led to a decrease in the risk of progression of pulmonary fibrosis. While using multivariate Cox regression analyses, acute/subacute onset of interstitial pneumonia (HR 3.231, 95%CI 1.936–5.392, *p* = 0.000), lower DLCO SB %pred (HR 6.435, 95%CI 4.072–10.017, *p* = 0.001), and the presence of DAD on HRCT (HR 8.679, 95%CI 1.974–38.157, *p* = 0.004) were associated with an increased risk of developing PPF. However, patients receiving triple therapy had a lower risk of progression to PPF (HR 0.322, 95%CI 0.115–0.899, *p* = 0.031) (Table 4). Interestingly, both univariate and multivariate analyses suggested that triple therapy reduced the risk of fibrosis progression.

**Table 4:** Cox proportional hazards analysis for PPF.

Variables	Univariate analysis		Multivariate analysis	
	HR (95% CI)	*p* value	HR (95% CI)	*p* value
Age	1.027 (1.007–1.047)	0.007		
Female	0.769 (0.511–1.159)	0.209		
Smokers	1.413 (0.915–2.183)	0.118		
BMI<18.5 (Kg/m^2^)	1.281 (0.405–4.055)	0.674		
BMI ≥ 25.0 (Kg/m^2^)	0.992 (0.632–1.559)	0.973		
Onset of ILD, acute/subacute	2.348 (1.559–3.538)	0.000	3.231 (1.936–5.392)	0.000
Proximal musculature weakness	0.768 (0.355–1.663)	0.503		
Gottron’s sign	0.897 (0.411–1.956)	0.785		
Mechanic’s hands	0.485 (0.224–1.051)	0.067		
Skin rash	0.894 (0.528–1.514)	0.677		
Photosensitivity	0.293 (0.092–0.927)	0.037		
Joint pain	0.864 (0.534–1.396)	0.550		
Anti-ARS antibodies	1.119 (0.722–1.735)	0.614		
Anti-MDA5 antibody	1.208 (0.729–2.003)	0.463		
Anti-Ro-52 antibody	1.039 (0.690–1.566)	0.853		
Anti-SSB antibody	3.244 (1.397–7.533)	0.006		
Anti-nuclear antibodies	0.895 (0.580–1.383)	0.617		
ANCA	3.022 (1.106–8.263)	0.031		
Anti-CCP antibody	2.166 (1.045–4.491)	0.038		
SIRI	1.095 (1.025–1.168)	0.008		
FVC, % pred	6.555 (4.682–9.175)	0.008		
DLCO, % pred	3.740 (1.667–8.388)	0.001	6.435 (4.072–10.017)	0.001
HRCT patterns		0.000		0.015
Unclassifiable IP^a^	Reference			
NSIP	1.176 (0.461–2.999)	0.735		
OP	1.127 (0.424–2.995)	0.811		
NSIP + OP	0.669 (0.078–5.762)	0.715		
UIP	1.782 (0.661–4.809)	0.254		
DAD	10.722 (3.652–31.474)	0.000	8.679 (1.974–38.157)	0.004
Triple therapies^b^	0.308 (0.123–0.776)	0.012	0.322 (0.115–0.899)	0.031

a Unclassifiable IP was as a reference.

b Triple therapies include corticosteroids, immunosuppressants and anti-fibrotic agents.

HR, hazard ratio; CI, confidence interval.

**Figure 2:**
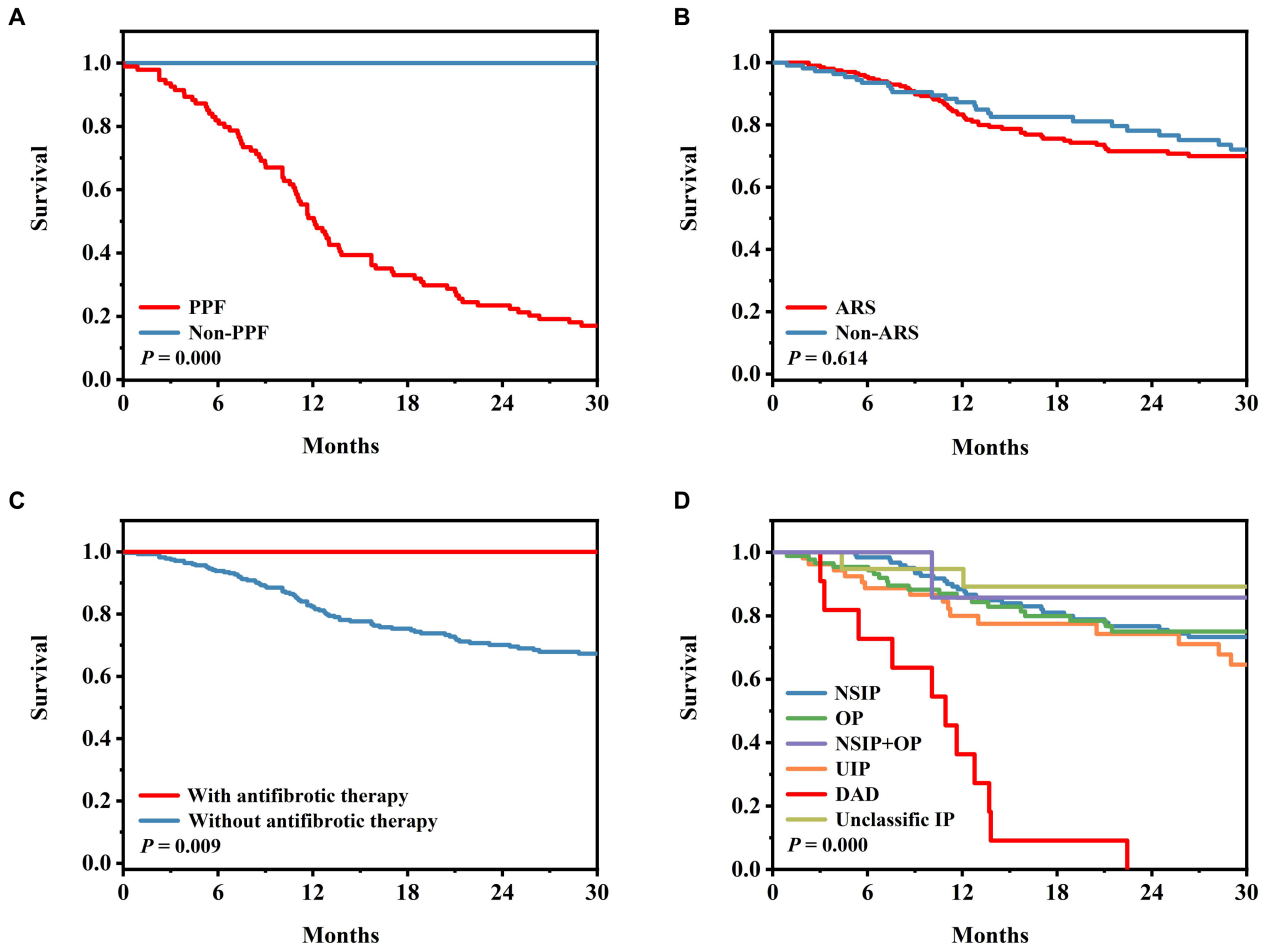
Survival in patients with PPF. **(A)** Survival according to the PPF group and non-PPF group (*p* = 0.000). **(B)** Survival according to anti-ARS and non-ARS MSA (*p* = 0.614). **(C)** Survival according to with or without anti-fibrotic treatment (*p* = 0.009). **(D)** Survival according to patterns on HRCT (*p* = 0.000). ARS, aminoacyl-tRNA synthase; DAD, diffuse alveolar damage; MSA, myositis-specific antibody; NSIP, nonspecific interstitial pneumonia; OP, organic pneumonia; PPF, progressive pulmonary fibrosis; UIP, usual interstitial pneumonia.

The median survival period for the PPF patients was 11.6 months, whereas, for the non-PPF patients, it was 31 months. Kaplan–Meier survival curves demonstrated a significantly lower survival rate in the PPF patients compared to the non-PPF patients (*p* = 0.000) (Figure 2A). The Kaplan–Meier curves for anti-ARS antibodies, triple therapy, and the presence of DAD on HRCT were consistent with the results of the Cox analysis (Figures 2B–D).

## Discussion

This study revealed that 30.6% of patients in the cohort developed PPF. The median survival for the entire cohort was 22 months, while PPF patients had a median survival of 11.6 months. PPF patients with MSA-IP exhibited predominant clinical characteristics such as dyspnea, lower baseline oxygenation index, and decreased DLCO SB% pred. Several factors were associated with an increased risk of developing PPF in MSA-IP patients, including acute/subacute onset of ILD, lower DLCO SB% pred. at initial diagnosis, and the presence of DAD pattern on HRCT. The study highlighted the potential of adding antifibrotic drugs (pirfenidone or nintedanib) to the treatment regimen of glucocorticoids combined with immunosuppressants in slowing down fibrosis progression. These findings underscore the importance of identifying PPF risk factors in MSA-IP patients and suggest that early intervention with antifibrotic drugs in combination with immunosuppressants could improve outcomes and extend survival.

PPF is a term used to describe the disease behavior of fibrotic ILDs, such as NSIP, fibrotic hypersensitivity pneumonitis, and CTD-associated ILD, indicating a prognosis similar to that of IPF (14, 15). The proportion of PPF varied widely in previous research, with approximately 15–50% of non-IPF ILD patients progressing to PPF (16–19). The prevalence of PPF in CTD-associated ILD depended on the underlying diagnosis, with an overall prevalence of 20–45% (17, 19, 20). Fibrosis progression occurs in up to half of patients with rheumatoid arthritis-related ILD (16, 17, 21, 22), and systemic sclerosis-associated ILD was observed in between 2 and 50% of PPF patients (17, 22, 23). The estimated incidence of PPF (approximately 30.6%) in this cohort aligns with previous research, highlighting regional and population variations in reported data.

This study demonstrated that patients with acute or subacute onset of ILD, lower baseline DLCO SB% pred. Values, and DAD patterns on HRCT were prone to developing PPF. To our knowledge, two predominant image patterns, namely NSIP and OP, are associated with myositis-associated ILD (7), while DAD is frequently observed at surgical lung biopsy or autopsy (24, 25). The proportion of DAD among PM/DM patients is approximately 20%, which is similar to systemic sclerosis and lower than rheumatoid arthritis (25). The clinical characteristics of DAD are consistent with acute respiratory distress syndrome in adults, which can be caused by pulmonary infection, sepsis, toxic inhalants, severe trauma, and drugs (24). Additionally, DAD is always observed in patients with acute/subacute IIM-ILD (26), and it may be the predominant pathological pattern in RPILD associated with anti-MDA5-related amyopathic dermatomyositis (27). The prognosis for this condition is extremely poor (26, 27). This may be associated with a worse prognosis in patients with^.^ Anti-MDA5 antibodies (28). Although few patients in this study underwent lung biopsies to confirm consistency, their clinical status was similar. The rapid progression of DAD and its poorer prognosis emphasize the need to identify and intervene for patients at risk of developing DAD. Increased serum Krebs von den Lungen-6 levels prior to the onset of respiratory symptoms may play a suggestive role in prognosis (29).

Pulmonary function serves as a key indicator of disease response and treatment efficacy. A study on pulmonary functions in PPF demonstrated an increased risk of developing PPF in patients with FVC <50% or DLCO SB% <35 (30). In addition to the aforementioned predictors, anti-Ro-52 and anti-MDA5 antibodies have also been recognized as risk factors for poorer prognosis (10, 11). MDA5 is encoded by the interferon induced helicase C domain containing protein 1 gene and functions as an intracellular sensor for double-stranded RNA viral replication intermediates or byproducts. Up to 90% of patients with anti-MDA5 antibodies can develop RPILD, which is associated with an extremely poor prognosis, with reported mortality rates between 50 and 80%, peaking within the first year or even the first 3 months (31, 32). Although one-third of anti-MDA5-positive patients in this study developed PPF, no statistically significant relationship was found.

In fibrotic ILDs other than IPF, there was no significant difference in the rate of decline in FVC between the nintedanib patients and the placebo patients, the effectiveness of nintedanib in reducing the decline in FVC may not be influenced by the use of immunomodulatory therapy (33). Therefore, combining nintedanib with immunosuppressive agents may be considered for patients with progressive fibrotic ILDs. Notably, the impact of nintedanib on lung function was found to be similar in patients with UIP-like patterns and other observed fibrosis patterns on HRCT (34, 35). The disease behavior of fibrotic ILDs associated with autoimmune diseases shares similarities with IPF, suggesting a potential application of antifibrotic drugs in this context, supported by some research evidence. Nintedanib can reduce the rate of decline in pulmonary function in patients with ILDs associated with systemic sclerosis while maintaining good safety and tolerability (36). However, the efficacy and safety of these drugs in patients with ILDs associated with IIM-ILD lack strong support from large-scale clinical trials. A study involving 170 patients with ILDs associated with autoimmune diseases (including only 2 with IIM-ILD) found that nintedanib reduced the rate of decline in FVC (37). A prospective open-label study focusing on anti-MDA5-associated clinically amyopathic dermatomyositis with RPILD showed no effect of pirfenidone on survival in patients with acute ILD (disease duration <3 months) (38). In patients with subacute ILD (disease duration 3–6 months), pirfenidone significantly improved survival (38). Additionally, nintedanib was found to reduce the incidence of RPILD and improve survival in patients with IIM-ILD (39). Regarding MSA-positive patients, our data suggested that triple therapy can delay fibrosis progression.

This study has several limitations that should be acknowledged. First, it is a retrospective cohort study, which limits control over data completeness and increases the risk of missing information. Second, being a single-center study, the results only represent the patients visiting this study hospital, and thus, they may not fully reflect the characteristics of the entire Chinese population. Third, 33.2% (102/307) of the patients underwent MSA revalidation within 1–3 years’ follow-up after the initial validation. The findings remained largely consistent, with only a minor subset of patients exhibiting negative results following treatment. Moreover, the therapy regimens applied in this study, which combined glucocorticoids and immunosuppressive drugs, cannot establish a definitive relationship with prognosis and fibrosis progression since there is no standardized treatment protocol regarding the type, dosage, indications, and duration of immunosuppressive therapy, especially for patients who are positive for MSA but do not meet the criteria for CTD.

In conclusion, this cohort study focused on MSA-IP patients and found that 30.6% of the enrolled patients developed PPF. Several predictors for PPF were identified, including acute/subacute onset of interstitial lung disease, a lower percentage of predicted DLCO SB%, and the presence of a DAD pattern on imaging. The addition of antifibrotic drugs (pirfenidone or nintedanib) to the treatment regimen of glucocorticoids combined with immunosuppressants showed the potential to slow down the progression of fibrosis. These findings underscore the importance of early identification of risk factors for PPF in MSA-IP patients and suggest that timely intervention with antifibrotic drugs in combination with immunosuppressants could improve outcomes and prolong survival. Further large-scale prospective studies are necessary to confirm these findings and provide more definitive evidence for the management of MSA-IP and PPF.

## Data availability statement

The raw data supporting the conclusions of this article will be made available by the authors, without undue reservation.

## Ethics statement

The studies involving humans were approved by Institutional Review Board of Beijing Chao-Yang Hospital. The studies were conducted in accordance with the local legislation and institutional requirements. Written informed consent for participation was not required from the participants or the participants’ legal guardians/next of kin because the study was a retrospective study, and the risk to the subjects was no greater than minimal risk. Waiving informed consent will not adversely affect the rights and health of the subjects. The privacy and personal identity information of the subjects will be effectively protected, therefore exempting the subjects from informed consent.

## Author contributions

HW: Writing – original draft. YW: Data curation, Writing – review & editing. DS: Data curation, Writing – review & editing. SY: Writing – review & editing. XD: Writing – review & editing. QY: Writing – review & editing.

## Figures and Tables

**Table:** 

ARS	aminoacyl-tRNA synthase
AISI	aggregate index of systemic inflammation
ANA	antinuclear antibody
BMI	body mass index
CTD	connective tissue disease
CI	confidence interval
CCP	cyclic citrullinated peptide
DM	dermatomyositis
DLCO	diffusing capacity of the lung for carbon monoxide
DAD	diffuse alveolar damage
dsDNA	double-stranded DNA
EJ	glycyl-tRNA synthetase
ESR	erythrocyte sedimentation rate
FVC	forced vital capacity
HRCT	high-resolution chest tomography
HR	hazard ratio
IIM	idiopathic inflammatory myopathy
ILD	interstitial lung disease
Ig	immune globulin
Jo-1	histidyl-tRNA synthetase
MSA	myositis-specific antibody
MDA	melanoma differentiation-associated gene
MPO-ANCA	myeloperoxidase-antineutrophil cytoplasmic antibody
MLR	monocyte/lymphocytes
NXP	nuclear matrix protein
NSIP	non-specific interstitial pneumonia
NLR	neutrophils/lymphocytes
OJ	isoleucyl-tRNA synthetase
OI	oxygenation index
OP	organic pneumonia
PPF	progressive pulmonary fibrosis
PM	polymyositis
PL-7	threonyl-tRNA synthetase
PL-12	alanyl-tRNA synthetase
PAH	pulmonary hypertension
PaO_2_/FiO_2_	partial pressure of oxygen
PLR	platelets/lymphocytes
PR3-ANCA	anti-proteinase 3-antineutrophil cytoplasmic antibody
RF	rheumatoid factor
RDW	red cell distribution width
RPILD	rapidly progressive interstitial lung disease
SRP	signal recognition particle
SAE	small ubiquitin-like modifier activating enzyme
SIRI	systemic inflammatory response index
TIF	transcriptional intermediary factor
UIP	usual interstitial pneumonia
WBC	white blood cell
