# Supplementary material for: Progressive pulmonary fibrosis in myositis-specific antibody-positive interstitial pneumonia: a retrospective cohort study
Source: Front Med (Lausanne). 2024 Jan 11;10:1325082. doi: 10.3389/fmed.2023.1325082 (PMC10808296; doi:10.3389/fmed.2023.1325082)
Supplement: Supplementary file 1 [file Data_Sheet_1.docx]

**Supplementary Material**

Progressive pulmonary fibrosis in myositis-specific antibody-positive interstitial pneumonia: a retrospective cohort study

Huijuan Wang^1^, Yuanying Wang^1^, Di Sun^1^, Shiwen Yu^1,2^, Xuqin Du^1,2^, Qiao Ye^1,2*^

^1^ Clinical Center for Interstitial Lung Diseases, Beijing Institute of Respiratory Medicine, Beijing Chao-Yang Hospital, Capital Medical University, Beijing, China

^2^ Department of Occupational Medicine and Toxicology, Beijing Chao-Yang

Hospital, Capital Medical University, Beijing, China

Corresponding Author

Prof. Dr. Qiao Ye，yeqiao_chaoyang@sina.com, ORCID ID: 0000-0002-0932-0487

Contents

| Materials and methods supplement | 1-2 |
| --- | --- |
| Table S1- MSA subtypes in the patients with progressive pulmonary fibrosis compared with non-progressive pulmonary fibrosis of MSA-IP | 3 |
| Table S2- HRCT patterns in the patients with progressive pulmonary fibrosis compared with in the non-progressive pulmonary fibrosis of MSA-IP | 4 |
|  |  |

Materials and methods

*MSA assay*

Venous blood was collected from patients, and 3-5 ml of serum was centrifuged and stored at-80℃. MSA was determined qualitatively using an immunoblotting method. The MSA subtypes measured included anti-ARS antibody, anti-SRP antibody, anti-Mi-2 antibody, anti-MDA5 antibody, anti-TIF1γ antibody, anti-NXP2 antibody, and anti-SAE antibody. The anti-ARS antibodies include anti-Jo-1, anti-PL-7, anti-PL-12, anti-OJ, anti-EJ et al. The kit was provided by Shenzhen Avalon Biotechnology Co. Ltd. and was operated strictly according to the instructions.

*Laboratory findings*

The derivative blood cell counts inflammation indexes included the monocyte to lymphocyte ratio (MLR, monocyte/lymphocytes), neutrophils to lymphocytes ratio (NLR, neutrophils/lymphocytes), platelets to lymphocytes ratio (PLR, platelets/lymphocytes), systemic inflammatory response index (SIRI, neutrophils × monocytes/lymphocytes), aggregate index of systemic inflammation (AISI, neutrophils × platelets × monocytes/lymphocytes).

*Definitions*

Patients were classified into three groups based on smoking history: non-smokers, former smokers (quit smoking for ≥12 months), and smokers (current smokers or those who quit smoking for less than 12 months). Patients who fulfilled the following criteria within 3 months from the onset of respiratory symptoms were considered acute/subacute ILD onset: 1) dyspnea: including chest tightness and shortness of breath; 2) hypoxemia, and 3) HRCT/Chest radiograph showing ground glass opacity or solid shadows. Chronic ILD onset was defined as >3 months from the onset of respiratory symptoms to the first visit. The treatment options include single glucocorticoid therapy, combination therapy of glucocorticoids with immunosuppressants, and triple therapy (glucocorticoids with immunosuppressants and antifibrotic drugs). The immunosuppressants mainly include azathioprine, cyclosporine, tacrolimus, cyclophosphamide, sulfasalazine, methotrexate, and hydroxychloroquine; antifibrotic drugs include nintedanib and pirfenidone.

*Sample size*

The main factor considered to influence the sample size was the prevalence of progressive pulmonary fibrosis in patients with fibrotic MSA-IP. Considering that the prevalence of progressive fibrosis is difficult to determine, it can only be estimated by retrospective analysis at this time. After reviewing the relevant literature, it was found that the prevalence of PPF in CTD-ILD ranged from 20% to 45%,^1-3^ which is 40% of IIM-ILD patients could develop PPF,^1^ and the proportion of patients with lost to follow-up was 10% in both groups. The total sample size of 307 patients (94 in the PPF group and 213 in the non-PPF group) was considered sufficient to detect the risk ratio (hazard ratio, HR) of fibrosis progression at a significance level of 0.05 and test effectiveness of >90%.

References

1. Hambly N, Farooqi MM, Dvorkin-Gheva A, Donohoe K, Garlick K, Scallan C, et al. Prevalence and characteristics of progressive fibrosing interstitial lung disease in a prospective registry. Eur Respir J. (2022) (4):2102571. doi: 10.1183/13993003.02571-2021
2. Wang Y, Guo Z, Ma R, Wang J, Wu N, Fan Y, et al. Prognostic predictive characteristics in patients with fibrosing interstitial lung disease: a retrospective cohort study. Front Pharmacol. (2022) 13:924754. doi: 10.3389/fphar.2022.924754
3. Faverio P, Piluso M, De Giacomi F, Della Zoppa M, Cassandro R, Harari S, et al. Progressive Fibrosing Interstitial Lung Diseases: Prevalence and Characterization in Two Italian Referral Centers. Respiration. (2020) 99(10):838-845.

**Table S1.** MSA subtypes in the patients with progressive pulmonary fibrosis compared with non-progressive pulmonary fibrosis of MSA-IP

| MSA subtype | All | PPF | Non-PPF | T/U/χ^2^ | *P*-value |
| --- | --- | --- | --- | --- | --- |
| N | 307 | 94 | 213 |  |  |
| Anti-ARS, n (%) | 199 (64.8)^*^ |  |  |  |  |
| Anti Jo-1 | 67 (21.8) | 13 (13.8) | 54 (25.4) | 5.075 | 0.024 |
| Anti PL-7 | 46 (15.0) | 21 (22.3) | 25 (11.7) | 5.756 | 0.016 |
| Anti PL-12 | 27 (8.8) | 9 (9.6) | 18 (8.5) | 0.103 | 0.749 |
| Anti OJ | 10 (3.3) | 7 (7.4) | 3 (1.4) | 7.546 | 0.011 |
| Anti EJ | 51 (16.6) | 15 (16.0) | 36 (16.9) | 0.042 | 0.838 |
| Non-ARS MSA, n (%) | 108 (35.2)^#^ |  |  |  |  |
| Anti SRP | 20 (6.5) | 9 (9.6) | 11 (5.2) | 2.083 | 0.149 |
| Anti Mi-2α | 5 (1.6) | 4 (4.3) | 1 (0.5) | 5.834 | 0.032 |
| Anti Mi-2β | 20 (6.5) | 6 (6.4) | 14 (6.6) | 0.004 | 0.950 |
| Anti TIF1γ | 15 (4.9) | 5 (5.3) | 10 (4.7) | 0.055 | 0.815 |
| Anti MDA5 | 57 (18.6) | 19 (20.2) | 38 (17.8) | 0.243 | 0.622 |
| Anti NXP2 | 10 (3.3) | 2 (2.1) | 8 (3.8) | 0.549 | 0.729 |
| Anti SAE1 | 6 (2.0) | 1 (1.1) | 5 (2.3) | 0.561 | 0.671 |

Data were presented as n (%).

* The reason for the above phenomenon is that 2 patients simultaneously tested positive for 2 different anti-ARS antibodies (1 patient tested positive for anti-PL-7 and anti-EJ, and another patient for anti-Jo-1 and anti-OJ).

# The reason for the above phenomenon is that one patient simultaneously tested positive for diverse different non-ARS MSA antibodies.

Abbreviations: ARS, aminoacyl-transfer-RNA synthase; MSA, myositis-specific antibodies; IP, interstitial pneumonia; Jo-1, histidyl-tRNA synthetase; PL-7, threonyl-tRNA synthetase; PL-12, alanyl-tRNA synthetase; OJ, isoleucyl-tRNA synthetase; EJ, glycyl-tRNA synthetase; SRP, signal recognition particle; TIF, transcriptional intermediary factor; MDA, melanoma differentiation-associated gene; NXP, nuclear matrix protein; SAE, small ubiquitin-like modifier activating enzyme.

**Table S2.** HRCT patterns in the patients with progressive pulmonary fibrosis compared with non-progressive pulmonary fibrosis of MSA-IP

| Variables | All | PPF | Non-PPF | T/U/χ^2^ | *P*-value |
| --- | --- | --- | --- | --- | --- |
| N | 307 | 94 | 213 |  |  |
| NSIP, n (%) | 126 (41.0) | 38 (40.4) | 88 (41.3) | 0.001 | 0.977 |
| OP, n (%) | 88 (28.7) | 21 (22.3) | 67 (31.5) | 3.327 | 0.068 |
| NSIP+OP, n (%) | 10 (3.3) | 1 (1.1) | 9 (4.2) | 2.069 | 0.293 |
| UIP, n (%) | 53 (17.3) | 18 (19.1) | 35 (16.4) | 0.337 | 0.562 |
| DAD, n (%) | 11 (3.6) | 11 (11.7) | 0 (0.0) | 17.157 | 0.000 |
| Unclassifiable IP, n (%) | 19 (6.2) | 5 (5.3) | 14 (6.6) | 0.185 | 0.667 |

Data were presented as n (%).

Abbreviations: HRCT, high-resolution chest tomography; MSA, myositis-specific antibodies; IP, interstitial pneumonia; NSIP, nonspecific interstitial pneumonia; OP, organic pneumonia; UIP, usual interstitial pneumonia; DAD, diffuse alveolar damage.
